# Editorial: Soft computing and machine learning applications for healthcare systems

**DOI:** 10.3389/frai.2026.1778974

**Published:** 2026-02-23

**Authors:** Ayodele A. Adebiyi, Olawande Daramola, Serestina Viriri, Emmanuel Adetiba

**Affiliations:** 1 Department of Computer Science, Landmark University, Omu-Aran, Nigeria; 2 Department of Informatics, University of Pretoria, Pretoria, South Africa; 3 Department of Computer Science, University of KwaZulu-Natal, Durban, South Africa; 4 Department of Electrical and Information Engineering, Covenant University, Ota, Nigeria; 5 HRA, Faculty of Engineering and the Built Environment, Durban University of Technology, Durban, South Africa

**Keywords:** artificial intelligence, bioinformatics, health informatics, healthcare system, machine learning

The integration of soft computing and machine learning into healthcare systems is increasing due to their effectiveness and precision (Javaid et al., 2022; Abdelaziz et al., 2018). In recent years, machine learning has had a profound impact on healthcare systems, transforming them through accurate disease diagnosis, personalized treatment, streamlined hospital operations, and assistance in drug discovery (Rani et al., 2025). Clinicians, researchers, and patients alike have readily embraced machine learning and soft computing applications as essential tools in modern medicine.

This Research Topic provides a platform for researchers and practitioners to present the theoretical and practical applications of soft computing and machine learning to improve the efficiency and effectiveness of the healthcare sector. Soft computing incorporates a group of computational techniques that are based on Artificial Intelligence (AI) and natural selection that make it possible to quickly and effectively solve complex problems for which analytical formulations are not practical. Typical soft computing techniques include Artificial Neural Networks, Fuzzy logic, Evolutionary algorithms, Swarm intelligence, and other computational methods that are based on approximate reasoning and modeling.

Obagbuwa et al. presented a study on supervised machine learning for sentiment analysis of depression. Their study utilized a machine learning model and sentiment analysis techniques to predict the level of depression in advance in social media users' posts. Four machine learning models, namely Extreme Gradient Boosting (XGB) Classifier, Random Forest, Logistic Regression, and Support Vector Machine (SVM), were employed for the prediction task. The study's findings highlighted the potential of utilizing machine learning models and sentiment analysis techniques for early detection of depression in social media users. The effectiveness of the SVM model and the efficiency of the logistic regression model, in terms of execution time, suggest their suitability for practical implementation in real-world scenarios.

In their study, Asani et al. developed a web-based plant diagnosis application called the mobile-enabled Plant Diagnosis-Application (mPD-App). The mPD-App was proposed to serve as a valuable tool for farmers and agricultural stakeholders in Sub-Saharan Africa, enabling them to detect and diagnose plant diseases effectively and efficiently. A convolutional neural network (CNN) model was used, achieving an overall accuracy of 93.91%.

Morapedi and Obagbuwa presented a study on predicting air pollution particulate matter (PM2.5) in South African cities using machine learning techniques. The study entailed the use of machine learning techniques such as CatBoost Regression, Extreme Gradient Boosting Regressor, Random Forest Classifier, Logistic Regression, Support Vector Machine, K-Nearest Neighbor, and Decision Tree to identify air pollution in terms of time, cost, and efficiency in different scenarios so that the system could select the optimal solution for their needs. Their findings showed that CatBoost Regressor and Extreme Gradient Boosting Regressor predict the latest PM2.5 concentrations for South African cities with recording stations using past-dated recordings, while Random Forest Classifier, Logistic Regression, Support Vector Machine, K-Nearest Neighbor, and Decision Tree predict the Air Quality Index status for South African cities.

In their study, Malik et al. explored dermoscopic structures for melanoma lesion classification. The authors explored AI's ability to discern melanoma from benign lesions using features of size, color, and shape. Tests with artificial and natural variations revealed a notable decline in accuracy, emphasizing the necessity of additional information, such as dermoscopic structures. The methodology used Transformers and CNN-based models to classify these images based on dermoscopic structures. Classification results were validated using feature visualization. To assess model susceptibility to image variations, classifiers were evaluated on test sets of original, duplicated, and digitally modified images. Additionally, testing was conducted on ISIC 2016 images. The study focused on three dermoscopic structures that are crucial for melanoma detection: Blue-white veils, dots/globules, and streaks. When evaluating model performance, adding convolutions to Vision Transformers was highly effective in achieving up to 98% accuracy. CNN architectures such as VGG-16 and DenseNet-121 reached 50–60% accuracy, performing best with features other than dermoscopic structures. Vision Transformers without convolutions exhibited reduced accuracy on diverse test sets, revealing their brittleness. OpenAI Clip, a pre-trained model, performed consistently well across various test sets. A mitigation method involving extensive data augmentation during training and 23 transformed duplicates during testing sustained accuracy and addressed brittleness.

Grassi et al. presented a study on enhanced sleep staging with artificial intelligence: a validation study of software for sleep scoring. The study extensively investigated Manual Sleep Staging (MSS), which uses polysomnography, and STAGER, a software program based on a machine learning algorithm that performs automatic sleep staging using only ECG signals from polysomnography. The findings revealed several agreement statistics between automatic sleep staging (ASS) and MSS, and among different MSSs. Their differences were then calculated. Bootstrap resampling was used to calculate 95% confidence intervals and the statistical significance of the differences. STAGER's ASS was most comparable to, or statistically significantly better than, MSS, except for a partial reduction in the positive percent agreement in the wake stage. These promising results indicate that the STAGER software can accurately perform ASS of inpatient polysomnographic recordings in comparison with MSS.

Ferhi et al. developed an enhanced, symptom-based health checker with a comprehensive machine learning approach that includes clinical vignettes and benchmarking. Their study focused on evaluating and optimizing machine learning models using a dataset of 10 diseases and 9,572 samples. The authors selected and optimized the following models: Decision Tree, Random Forest, Naïve Bayes, Logistic Regression, and K-Nearest Neighbor. The evaluation metrics used were accuracy and F1 scores. ROC-AUC curves and precision-recall curves were also used to assess model performance. The ROC-AUC curves revealed that model performance improves with increasing complexity. Precision-recall curves were particularly useful for evaluating model sensitivity in imbalanced dataset scenarios. The clinical vignettes demonstrated the robustness of the models in providing accurate diagnoses.

Khan and O'Sullivan presented a study comparing the diagnostic ability of large language models (LLMs) in challenging clinical cases. The study compared the different performance characteristics of common LLMs in solving complex clinical cases and assessed the utility of a novel tool to grade LLM output. The authors performed a comparative analysis of three LLM models—Bing, ChatGPT, and Gemini—across a diverse set of clinical cases as presented in the New England Journal of Medicine's case series. The results revealed that the models perform differently when presented with identical clinical information, with Gemini performing best. The grading tool had low interobserver variability and proved to be a reliable tool for grading LLM clinical output.

Huang et al. proposed an unsupervised machine learning model for the detection of anomalous volumetric modulated arc therapy (VMAT) plans for lung cancer patients. In the study, a multi-task AutoEncoder (AE) was proposed to automate VMAT anomaly detection for lung cancer patients. Among the four tested AE models, the proposed multi-task AE model achieved the highest values in AUC (0.964), accuracy (0.821), precision (0.471), and F1 score (0.632), and the lowest value in FPR (0.206). Using two-dimensional (2D) feature maps, the proposed multi-task AE model can effectively detect anomalies in radiotherapy plans for lung cancer patients. Compared to the other existing AE models, the multi-task AE is more accurate and efficient.

Liu et al. investigated modeling disagreement in automatic data labeling for semi-supervised learning in clinical natural language processing. The authors investigated the quality of uncertainty estimates from a range of current state-of-the-art predictive models applied to the problem of observation detection in radiology reports. The problem remains understudied for Natural Language Processing in the healthcare domain. Their findings revealed that Gaussian Processes (GPs) provide superior performance in quantifying the risk of three uncertainty labels based on the negative log predictive probability (NLPP) evaluation metric and mean maximum predicted confidence levels (MMPCL), while retaining strong predictive performance.

Wang et al. applied machine learning to intelligent systems with a knowledge graph-enhanced ophthalmic contrastive learning approach using a clinical profile prompt. Their study investigated the application of machine learning techniques by integrating knowledge graphs with contrastive learning and utilizing “clinical profile” prompts to refine the performance of the ophthalmology-specific large language model, MeEYE, which is built on the CHATGLM3-6B architecture. The study employed a novel methodological framework that incorporates domain-specific knowledge through knowledge graphs and enhances feature representation using contrastive learning. The MeEYE model was fine-tuned with structured clinical knowledge, enabling it to better distinguish subtle yet significant ophthalmic features. The experimental findings demonstrated that integrating knowledge graphs and contrastive learning into the MeEYE model significantly improves both diagnostic accuracy and model interpretability. Comparative analyses against baseline models reveal that the proposed approach enhances the identification of ophthalmic conditions with greater precision and clarity.

Finally, Hsu et al. explored pivotal tongue diagnostic variables between patients with chronic kidney disease and healthy participants. Their study examined the relationship between tongue characteristics and chronic kidney disease (CKD) severity using an automatic tongue diagnosis system (ATDS), which captures tongue images non-invasively to provide objective diagnostic information. A cross-sectional, case-control study was conducted from 1 July 2019 to 31 December 2021. Participants were divided into three groups based on their estimated glomerular filtration rate (eGFR): control (eGFR > 60 mL/min/1.732), CKD stage 3 (30 ≤ eGFR < 60 mL/min/1.732), and CKD stage 4–5 (eGFR < 30 mL/min/1.732). Tongue images were analyzed using ATDS to extract nine primary features: tongue shape, color, fur, saliva, fissures, ecchymosis, tooth marks, and red dots. The study revealed significant differences in fur thickness, tongue color, ecchymosis amount, and saliva among the three groups. Ordinal logistic regression analysis indicated that pale tongue color (OR: 2.107, *P* < 0.001), bluish tongue color (OR: 2.743, *P* = 0.001), yellow fur (OR: 3.195, *P* < 0.001), wet saliva (OR: 2.536, *P* < 0.001), and ecchymoses (OR: 1.031, *P* = 0.012) are significantly associated with increased CKD severity.

The study consists of the contributions of each author in the research topic. The limitations of the studies included are the use of only a few soft computing techniques. The contributions reveal a gap in the minimal amount of healthcare systems applications with soft computing and machine learning. A future research priority is fusing machine learning techniques for the effective diagnosis of diseases.
